# Psychotropic Medication Use Before and During COVID-19: A Population-Wide Study

**DOI:** 10.3389/fphar.2022.886652

**Published:** 2022-04-27

**Authors:** Christine Leong, Kaarina Kowalec, Sherif Eltonsy, James M. Bolton, Murray W. Enns, Qier Tan, Marina Yogendran, Dan Chateau, Joseph A. Delaney, Jitender Sareen, Jamison Falk, Rae Spiwak, Sarvesh Logsetty, Silvia Alessi-Severini

**Affiliations:** ^1^ College of Pharmacy, Rady Faculty of Health Sciences, University of Manitoba, Winnipeg, MB, Canada; ^2^ Department of Psychiatry, Max Rady College of Medicine, Rady Faculty of Health Sciences, University of Manitoba, Winnipeg, MB, Canada; ^3^ Department of Medical Epidemiology and Biostatistics, Karolinska Institutet, Solna, Sweden; ^4^ Department of Community Health Sciences, Max Rady College of Medicine, Rady Faculty of Health Sciences, University of Manitoba, Winnipeg, MB, Canada; ^5^ Department of Psychology, University of Manitoba, Winnipeg, MB, Canada; ^6^ Manitoba Centre for Health Policy, Max Rady College of Medicine, Rady Faculty of Health Sciences, University of Manitoba, Winnipeg, MB, Canada; ^7^ Research School of Population Health, College of Health and Medicine, Australian National University, Canberra, ACT, Australia; ^8^ Department of Epidemiology, University of Washington, Seattle, WA, United States; ^9^ Department of Surgery, Max Rady College of Medicine, Rady Faculty of Health Sciences, University of Manitoba, Winnipeg, MB, Canada

**Keywords:** psychotropic drugs, COVID-19, pandemic, drug utilization, population-wide study

## Abstract

**Background:** The coronavirus disease 2019 (COVID-19) pandemic and public health measures that took place have led to concerns regarding mental health and receipt of psychotropic medications. We aimed to study the changes in psychotropic medication dispensation rates before and during the COVID-19 pandemic in the general population.

**Methods:** Administrative health data from the Canadian province of Manitoba was used to describe the quarterly incidence and prevalence of antipsychotics, antidepressants, and anxiolytic/sedative-hypnotics from January 1, 2015 to December 31, 2020. Individuals who received at least one prescription within each quarter were considered exposed to the medication. The denominator was the total population within each quarter. Incidence was defined as no receipt of medication in the 3 years prior to the quarter of interest. Autoregression models for time series data plus indicator variables were used to compare each quarter of 2020 after public health measures were implemented in March 2020 in relation to the expected trend. Analyses were stratified by age and sex.

**Results:** There were 1,394,885 individuals in the first quarter of 2020, with a mean (SD) age of 38.9 (23.4) years, 50.3% were female, and 36.1% had a psychiatric diagnosis in the previous 5 years. A significant decrease was observed for incident antidepressant use (*p* < 0.05 for both sexes and all age groups except for those 65 years and older) and anxiolytic use (*p* < 0.05 for both sexes and all age groups except 80 years and older) in the second quarter (April-June) of 2020 compared to the expected trend. Females and those aged 40 years and older had a significantly higher incidence of antidepressant and antipsychotic use in the final quarter of 2020 compared to the expected trend (*p* < 0.05).

**Conclusion:** Our findings indicate a decrease in new prescriptions for antidepressants and anxiolytics in the 3 months after COVID-19 in-person restrictions were first implemented. We then observed an increase in the new use of antidepressants and antipsychotics at the end of 2020, in females and people aged 40 years and older, with the highest rates of use in the population 80 years and older.

## Introduction

The mental health and wellbeing of individuals during the coronavirus disease 2019 (COVID-19) pandemic and after public health measures took place has been at the forefront of concerns related to the pandemic (Canadian Centre on Substance Use and Addiction, 2020; Holmes et al., 2020; Gunnell et al., 2020; Vigo et al., 2020; Canadian Mental Health Association National Survey, 2020). National surveys in Canada reported increased anxiety, depression, and substance use (Canadian Mental Health Association National Survey, 2020; Mental Health Commission of Canada and Canadian Centre, 2021; Vindegaard and Benros, 2020), with 40% of Canadians reporting a decline in mental health since March 2020 (Mental Health Commission of Canada and Canadian Centre, 2021). Changes in financial circumstances, social isolation, and the health of family members were identified as the top three stressors related to the pandemic (Vindegaard and Benros, 2020; Mental Health Commission of Canada and Canadian Centre, 2021). Internationally, the rate of insomnia and symptoms of depression and anxiety increased during the initial months of the pandemic compared to the previous year (Li et al., 2020; Voitsidis et al., 2020; Kokou-Kpolou et al., 2020; The COVID-19 Healthcare Coalition Telehealth Impact Study Work Group, 2020). Understanding the mental health effects of COVID-19 and related public health measures has become an important research priority (Gunnell et al., 2020; Holmes et al., 2020). Examining trends of psychotropic medication prescribing provides important information on pandemic-related distress and health service use.

During the pandemic, the public has experienced restrictions to in-person healthcare visits (Supplementary Appendix SI), and these measures have shifted the way in which people were able to seek care. A higher rate of virtual visits in place of in-person for outpatient mental health care has been observed during the pandemic (Grekou et al., 2021). It is anticipated that such changes would have an impact on the prescribing of certain psychotropic medications, such as antidepressants, antipsychotics, and anxiolytic/sedative-hypnotics. Furthermore, it is uncertain whether there will be differences in the incidence and prevalence of psychotropic medication by age and sex. Previous reports have noted that females experienced greater challenges during the pandemic as a result of unemployment and unreliable childcare (Centre for Addiction and Mental Health, 2020; Thibaut and van Wijngaarden-Cremers, 2020; Statsitics Canada, 2021). In contrast, the mental health of older adults was found to be less affected by the pandemic compared to the younger population (Roos et al., 2005; Vahia et al., 2020). A shift in psychotropic medication prescribing can have implications on the health outcomes of patients. Identifying groups with greater incidence or prevalence of psychotropic medication is essential for gaining a better understanding of drug prescribing and need for targeted interventions to address potential mental health impacts of the pandemic.

Administrative data can provide rich information on the real-world effects of a pandemic on medication use. The objective of this study was to determine if the quarterly incidence and prevalence of psychotropic medication use changed from 2015 to 2020 in the general population and whether this differed by age and sex. At the time the study was conducted, we hypothesized that psychotropic medication incidence and prevalence would decrease in the second quarter of 2020 after the new restrictions to in-person visits took place followed by an increase in psychotropic use in the last quarter of 2020. We also hypothesized that females and younger adults will have higher incidence and prevalence in psychotropic medication use compared to males and other age groups, respectively, during the pandemic.

## Materials and Methods

### Data Source

This was a longitudinal whole population observational study using administrative health data from the Manitoba Population Research Data Repository located at the Manitoba Centre for Health Policy (MCHP). This repository, which has been used extensively for population-wide research (Roos and Nicol, 1999; Daumit et al., 2003), contains data on physician visits, hospitalizations, and medication dispensing that is, not restricted to age, income, or healthcare coverage, for all residents of Manitoba (a population of approximately 1.4 million). A significant strength of these data is that the Drug Program Information Network (DPIN) contains information on the strength, days supply, quantity, and date of prescription filled for all Manitoba residents regardless of age or drug coverage, except for medications received in the hospital and nursing stations. Physician claims data and hospital discharge abstracts provided information on contacts with the healthcare system and diagnoses using the International Classification of Diseases, Clinical Modification (ICD-9-CM or ICD-10-CA equivalent) codes. The Manitoba Health Insurance Registry provided demographic information on age, sex, and urban/rural residence at the beginning of each interval. Statistics Canada census files provided income quintile information. This study was approved by the Human Research Ethics Board of the University of Manitoba and the Manitoba Health Seniors and Active Living (MHSAL) Health Information Privacy Committee (HIPC). These factors give us the unique capacity to study populations often under-represented in administrative-based studies conducted in other jurisdictions.

### Population

All community-dwelling individuals not restricted by age living in Manitoba with at least 1 day of MHSAL coverage between January 1, 2015 and December 31, 2020 were included. For each quarter or year of interest, the denominator for the general population was the sum of individuals who were listed in the MHSAL registry for at least 1 day of coverage during that quarter or year. Manitoba residents who were dispensed ≥1 psychotropic or non-psychotropic medication within each quarter from January 1, 2015 to December 31, 2020 were identified as the population of psychotropic or non-psychotropic medication users, respectively. Data from 2015 to 2019 was included to allow us to account for underlying trends in utilization in the period prior to the pandemic. Those with a mental disorder during the study period were further described by the following categories: mood and/or anxiety, psychosis, schizophrenia, personality disorder, and substance use disorder using ICD codes previously used in research conducted at MCHP (See Supplementary Appendix SII for ICD codes) (Daumit et al., 2003; Brownell et al., 2012; Smith et al., 2013; Brownell et al., 2015; Chartier et al., 2015).

### Drug Exposure

All medications included in the analysis were identified using their Anatomic Therapeutic Classification (ATC) code (World Health Organization, 2021). Psychotropic medications included antidepressants (ATC N06A and N06CA), anxiolytic/sedative-hypnotics (including benzodiazepines and z-drug hypnotics, N05B, N05C, and N03AE01), and antipsychotic agents (N05A, except N05AN). Medication exposure was defined as at least one dispensation of the medication of interest within each calendar quarter (quarter 1 was January-March, quarter 2 was April-June, quarter 3 was July-September, and quarter 4 was October-December).

### Statistical Analyses

Demographic characteristics including age (≤18, 19–39, 40–64, 65–79, ≥80 years old), sex, region of residence, socioeconomic status [(SES) based on neighborhood income quintile] and psychiatric disorder type in the previous 5 years (mood/anxiety, psychosis, substance use disorder, personality disorder, schizophrenia) (MCHP Concept Dictionary, 2016) for the first quarter of 2020 were described using summary statistics.

The primary analysis described the quarterly prevalence and incidence rates of dispensed psychotropic medications from January 1, 2015 to December 31, 2020 overall and then stratified by age group and sex. Incident users were defined as those who had not been dispensed a medication from the drug class of interest in the 3 years prior to their first dispensation. The rate of dispensing of each drug class was determined for each quarter by counting the number of individuals dispensed a prescription for that medication class divided by the total number of individuals in that quarter for the general population and expressed as per 1,000 people in the general population per quarter.

Autoregression models for time series data plus indicator variables were used to examine rates of psychotropic medication use before and after the second quarter of 2020 using interrupted time series models with autocorrelation to look at quarterly incidence and prevalence. Of note, because the data show a unique fluctuation in quarterly rates after the time after public health restrictions took place in March, an indicator variable was used to determine if the quarterly rates of Q2, Q3, and Q4 of 2020 were significantly different from the secular trends in the model. A coefficient expressing the difference from expected from each of the three quarters of 2020 was reported. Statistical significance was set at *p* < 0.05. SAS statistical software (version 9.4, SAS Institute, Cary, NC) was used for all analyses.

## Results

The study population in the first quarter of each year ranged from 1,331,188 in 2015 to 1,394,885 in 2020. The demographic characteristics of the study population at the beginning of the first quarter of 2020 are shown in Table 1. The mean (SD) age was 38.9 (23.4) years, 50.3% were female, and 61.5% resided in an urban residence. There were 36.1% who were diagnosed with a mental disorder in the previous 5 years, with 29% having a history of mood or anxiety disorder.

**TABLE 1:** Demographics of the study population for the first quarter of 2020 (N = 1,394,885).

Demographic	Frequency (%)
Mean age (years)	38.9 (SD 23.4)
Age group (years)	
≤18	330,398 (23.7)
19–39	403,129 (28.9)
40–64	435,354 (31.2)
65–79	167,991 (12.0)
≥80	58,013 (4.2)
Female sex	701,368 (50.3)
Income quintile (1 = lowest; 5 = highest)	
Rural 1	107,078 (7.7)
Rural 2	107,838 (7.7)
Rural 3	108,083 (7.8)
Rural 4	107,114 (7.7)
Rural 5	105,369 (7.6)
Urban 1	169,395 (12.1)
Urban 2	168,661 (12.1)
Urban 3	169,218 (12.1)
Urban 4	169,994 (12.2)
Urban 5	170,216 (12.2)
Not found	11,919 (0.85)
Urban residence (Winnipeg/Brandon)	858,118 (61.5)
Psychiatric diagnosis in last 5 years	503,575 (36.1)
Mood/Anxiety	404,822 (29.0)
Psychosis	59,522 (4.3)
Substance use disorder	50,658 (3.6)
Personality disorder	9,025 (0.7)
Schizophrenia	8,806 (0.6)

### Antidepressants

**FIGURE 1:**
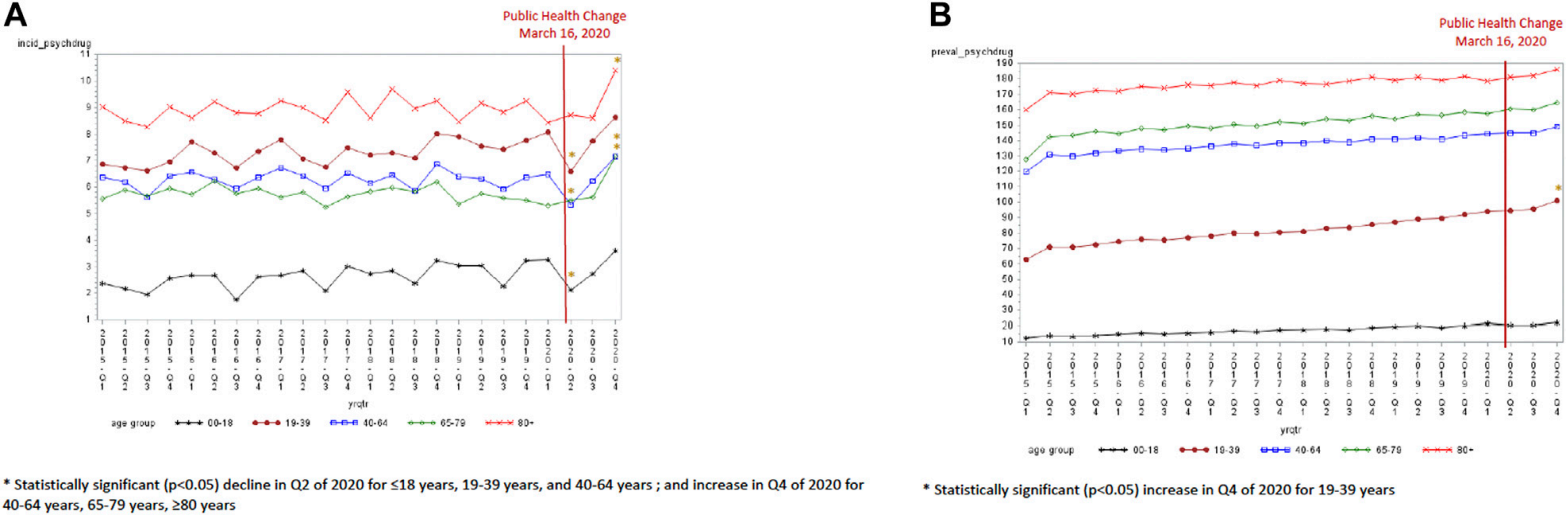
**(A)** Antidepressant incidence by age group (per 1,000). **(B)** Antidepressant prevalence by age group (per 1,000).

Overall, the incidence of antidepressant dispensations ranged from 5.6 per 1,000 in quarter 1 of 2015 to 6.9 per 1,000 in the final quarter of 2020, while the prevalence of antidepressant use increased from 79.9 per 1,000 in the first quarter of 2015 to 108.6 per 1,000 in the final quarter of 2020 (Supplementary Appendix SIV). The incidence of antidepressant use was lowest in the second quarter of 2020 (5.09 per 1,000) and the highest in the last quarter of 2020 (6.87 per 1,000). The groups aged ≤18 years [−0.97, standard error (SE) 0.38, *p* = 0.02], 19–39 years (−1.27, SE 0.38, *p* = 0.0031), and 40–64 years (−1.04, SE 0.34, *p* = 0.006) experienced a statistically significant decline in incident antidepressant use in the second quarter of 2020 compared to the expected trend (Figure 1A and Table 2). A statistically significant increase in the incidence of antidepressant use was seen in the last quarter of 2020 for those 40 years and older (40–64 years old: 0.77, SE 0.34, *p* = 0.04; 65–79 years old: 1.59, SE 0.29, *p* < 0.0001; and ≥80 years old: 1.33, SE 0.44, *p* = 0.007). An increase in the prevalence of antidepressant use was observed in the last quarter of 2020 relative to the expected trend for the population aged 19–39 years (4.77, SE 1.80, *p* = 0.016) only (Figure 1B and Table 3).

**TABLE 2:** Coefficient estimate (standard error, SE) and *p*-value measuring the difference in incidence rates from the expected trend in each quarter of 2020 after public health restrictions were implemented.

Parameter	Q2 (Apr-June 2020)	3 (Jul-September 2020)	Q4 (Oct-December 2020)
**Antidepressant**			
≤18 years	−0.97 (0.38, *p* = 0.02)*	−0.41 (0.39, *p* = 0.30)	0.42 (0.39, *p* = 0.29)
19–39 years	−1.27 (0.38, *p* = 0.003)*	−0.15 (0.38, *p* = 0.70)	0.67 (0.39, *p* = 0.10)
40–64 years	−1.04 (0.34, *p* = 0.006)*	−0.16 (0.34, *p* = 0.64)	0.77 (0.35, *p* = 0.04)*
65–79 years	−0.091 (0.28, *p* = 0.75)	0.044 (0.28, *p* = 0.88)	1.59 (0.29, *p* < 0.0001)*
≥80 years	−0.31 (0.42, *p* = 0.47)	−0.43 (0.43, *p* = 0.32)	1.33 (0.44, *p* = 0.007)*
Female	−1.14 (0.35, *p* = 0.004)*	−0.097 (0.36, *p* = 0.79)	1.25 (0.36, *p* = 0.003)*
Male	−0.76 (0.26, *p* = 0.009)*	−0.32 (0.27, *p* = 0.25)	0.29 (0.27, *p* = 0.29)
**Anxiolytic/Sedative-Hypnotic**			
≤18 years	−0.41 (0.11, *p* = 0.002)*	0.11 (0.12, *p* = 0.35)	−0.23 (0.12, *p* = 0.06)
19–39 years	−1.01 (0.25, *p* = 0.0006)*	−0.35 (0.25, *p* = 0.18)	−0.93 (0.25, *p* = 0.002)*
40–64 years	−1.10 (0.34, *p* = 0.005)*	−0.72 (0.35, *p* = 0.05)	−1.11 (0.35, *p* = 0.005)*
65–79 years	−0.85 (0.30, *p* = 0.01)*	−0.53 (0.31, *p* = 0.10)	−0.74 (0.31, *p* = 0.03)*
≥80 years	−0.11 (0.53, *p* = 0.85)	−0.08 (0.54, *p* = 0.89)	−0.061 (0.54, *p* = 0.91)
Female	−1.04 (0.19, *p* < 0.0001)*	−0.35 (0.19, *p* = 0.08)	−0.93 (0.19, *p* = 0.0001)*
Male	−0.64 (0.23, *p* = 0.01)*	−0.38 (0.23, *p* = 0.12)	−0.60 (0.23, *p* = 0.019)*
**Antipsychotic**			
≤18 years	−0.045 (0.11, *p* = 0.68)	−0.075 (0.11, *p* = 0.50)	0.17 (0.11, *p* = 0.15)
19–39 years	−0.20 (0.12, *p* = 0.12)	−0.0064 (0.13, *p* = 0.96)	0.13 (0.13, *p* = 0.31)
40–64 years	−0.16 (0.11, *p* = 0.16)	−0.088 (0.11, *p* = 0.43)	0.29 (0.11, *p* = 0.02)*
65–79 years	0.18 (0.17, *p* = 0.31)	0.25 (0.18, *p* = 0.17)	0.82 (0.18, *p* = 0.0002)*
≥80 years	0.10 (0.37, *p* = 0.01)*	0.71 (0.37, *p* = 0.07)*	1.88 (0.38, *p* < 0.0001)*
Female	−0.05 (0.12, *p* = 0.64)	0.06 (0.12, *p* = 0.60)	0.52 (0.12, *p* = 0.0004)*
Male	−0.06 (0.11, *p* = 0.56)	−0.03 (0.11, *p* = 0.77)	0.17 (0.11, *p* = 0.13)

**TABLE 3:** Coefficient estimate (standard error, SE) and *p*-value measuring the difference in prevalence rates from the expected trend in each quarter of 2020 after public health restrictions were implemented.

Parameter	Q2 (Apr-June 2020)	Q3 (Jul-September 2020)	Q4 (Oct-December 2020)
**Antidepressant**			
≤18 years	−0.66 (0.53, *p* = 0.23)	−1.03 (0.53, *p* = 0.07)	0.28 (0.54, *p* = 0.61)
19–39 years	0.80 (1.76, *p* = 0.65)	0.66 (1.78, *p* = 0.72)	4.77 (1.80, *p* = 0.02)*
40–64 years	−0.87 (2.51, *p* = 0.73)	−1.70 (2.54, *p* = 0.51)	1.66 (2.57, *p* = 0.53)
65–79 years	−0.17 (3.47, *p* = 0.96)	−1.84 (3.52, *p* = 0.61)	1.64 (3.56, *p* = 0.65)
≥80 years	−2.25 (2.91, *p* = 0.45)	−1.85 (2.94, *p* = 0.54)	1.54 (2.98, *p* = 0.61)
Female	−0.17 (2.37, *p* = 0.94)	−0.21 (2.40, *p* = 0.93)	4.07 (2.43, *p* = 0.11)
Male	−0.66 (1.10, *p* = 0.56)	−1.03 (1.12, *p* = 0.37)	0.80 (1.13, *p* = 0.49)
**Anxiolytic/Sedative-Hypnotic**			
≤18 years	−0.66 (0.23, *p* = 0.009)*	0.21 (0.23, *p* = 0.38)	−0.28 (0.23, *p* = 0.25)
19–39 years	−2.04 (1.31, *p* = 0.13)	−1.73 (1.32, *p* = 0.21)	−2.33 (1.34, *p* = 0.098)
40–64 years	−3.47 (2.75, *p* = 0.22)	−5.44 (2.78, *p* = 0.065)	−5.20 (2.82, *p* = 0.08)
65–79 years	−3.26 (4.72, *p* = 0.50)	−6.17 (4.78, *p* = 0.21)	−5.88 (4.84, *p* = 0.24)
≥80 years	−0.94 (4.45, *p* = 0.84)	−4.75 (4.51, *p* = 0.31)	−0.93 (4.57, *p* = 0.84)
Female	−2.57 (2.27, *p* = 0.27)	−3.40 (2.29, *p* = 0.15)	−3.46 (2.32, *p* = 0.15)
Male	−1.86 (1.36, *p* = 0.21)	−2.20 (1.37, *p* = 0.13)	−2.22 (1.39, *p* = 0.13)
**Antipsychotic**			
≤18 years	0.30 (0.17, *p* = 0.10)	0.18 (0.17, *p* = 0.31)	0.51 (0.18, *p* = 0.01)*
19–39 years	0.33 (0.29, *p* = 0.26)	0.50 (0.29, *p* = 0.10)	1.18 (0.30, *p* = 0.0008)*
40–64 years	0.27 (0.25, *p* = 0.28)	0.15 (0.25, *p* = 0.56)	0.62 (0.25, *p* = 0.02)*
65–79 years	0.28 (0.34, *p* = 0.42)	0.85 (0.35, *p* = 0.02)*	1.43 (0.35, *p* = 0.0006)*
≥80 years	1.96 (0.98, *p* = 0.06)	2.70 (0.99, *p* = 0.01)*	3.70 (1.00, *p* = 0.002)*
Female	0.50 (0.28, *p* = 0.09)	0.81 (0.28, *p* = 0.01)	1.47 (0.29, *p* < 0.0001)*
Male	0.22 (0.24, *p* = 0.36)	0.16 (0.24, *p* = 0.51)	0.52 (0.25, *p* = 0.046)*

**FIGURE 2:**
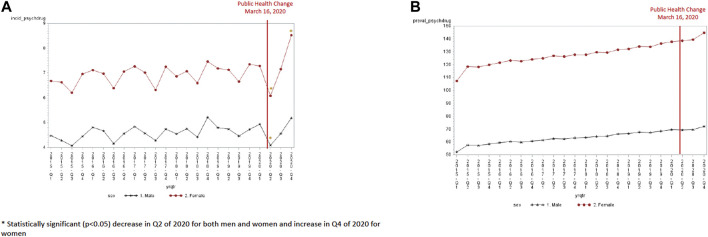
**(A)** Antidepressant incidence by sex (per 1,000). **(B)** Antidepressant prevalence by sex (per 1,000).

Both males and females experienced a decline in antidepressant incidence in the second quarter of 2020 (4.09 per 1,000 for males and 6.08 per 1,000 for females) (Figure 2A). This decline in quarter 2 was significantly lower than the expected trend for both males (−0.76, SE 0.26, *p* = 0.009) and females (−1.14, SE 0.35, *p* = 0.004). However, females experienced a greater increase in antidepressant incidence than males in the last quarter of 2020 (8.52 per 1,000 in quarter 4 of 2020 for females and 5.19 per 1,000 in quarter 4 of 2020 for males). This increase in quarter 4 was significantly higher than the expected trend for females (1.25, SE 0.36, *p* = 0.003) but not for men (0.29, SE 0.27, *p* = 0.29). The prevalence of antidepressant use was higher for females and males in the last quarter of 2020 (144.7 per 1,000 for females versus 72.1 per 1,000 for males). However, the increase in prevalence in the final quarter of 2020 was not significantly different than the expected trend (4.07, SE 2.4, *p* = 0.111 for females and 0.80, SE 1.13, *p* = 0.50 for men) (Table 3).

### Anxiolytic/Sedative-Hypnotics

**FIGURE 3:**
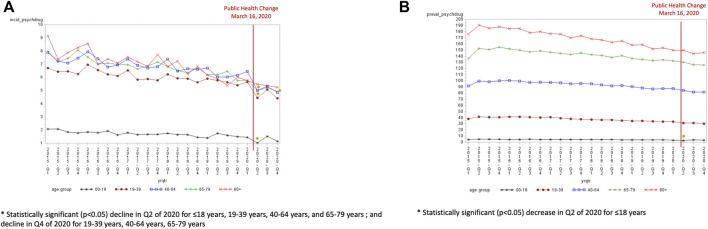
**(A)** Anxiolytic/sedative-hypnotic incidence by age group (per 1,000). **(B)** Anxiolytic/sedative-hypnotic prevalence by age group (per 1,000).

Overall, the incidence (6.2 per 1,000 in Q1 of 2015 to 3.9 per 1,000 in Q4 of 2020) and prevalence (63.5 per 1,000 in Q1 of 2015 to 56.3 per 1,000 in Q4 of 2020) use of anxiolytic/sedative-hypnotics declined from 2015 to 2019 (Supplementary Appendix SIV). All age groups experienced a decline in anxiolytic/sedative-hypnotic incidence in quarter 2 of 2020 (Figure 3A). The decline in quarter 2 was significant for all age groups except for those 80 years and older (−0.06, SE 0.54, *p* = 0.912) when compared to the expected trend. The incidence of anxiolytic/sedative-hypnotics were also significantly lower than the expected trend in the final quarter of 2020 for those aged 19–39 years (−0.93, SE 0.25, *p* = 0.002), 40–64 years (−1.11, SE 0.35, *p* = 0.005), and 65–79 years (−0.74, SE 0.31, *p* = 0.027). Only the population aged <18 years experienced a significant decline in prevalence in Q2 of 2020 compared to the expected trend (−0.66, SE 0.23, *p* = 0.009) (Figure 3B).

**FIGURE 4:**
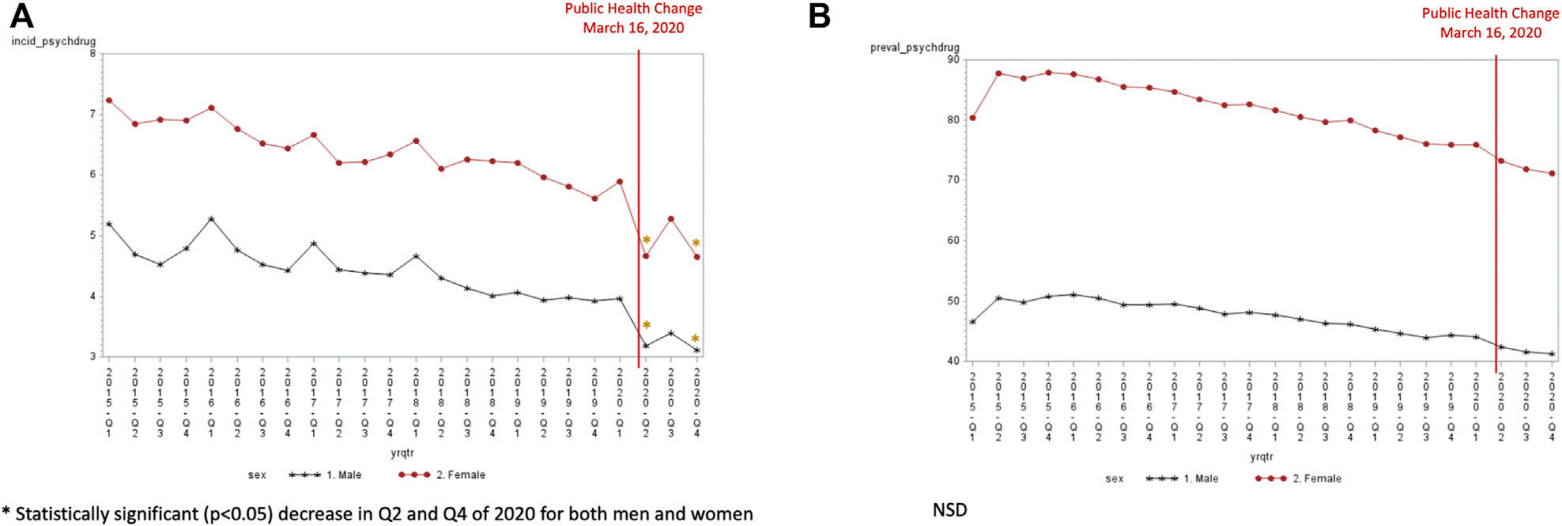
**(A)** Anxiolytic/sedative-hypnotic incidence by sex (per 1,000). **(B)** Anxiolytic/sedative-hypnotic prevalence by sex (per 1,000).

Both males and females experienced a decline in anxiolytic/sedative-hypnotic incidence in the second quarter of 2020 (3.18 per 1,000 for men and 4.66 per 1,000 for females) (Figure 4A). This decline in quarter 2 of 2020 was significantly lower than the expected trend for both males (−0.64, SE 0.23, *p* = 0.011) and females (−1.04, SE 0.19, *p* < 0.0001). The decline was also significantly lower in quarter 4 of 2020 than the expected trend for both males (−0.60, SE 0.23, *p* = 0.019) and females (−0.93, SE 0.19, *p* = 0.0001). The prevalence of anxiolytic/sedative-hypnotics were not significantly different in quarters 2 to 4 of 2020 compared to the expected trend for both males and females (Figure 4B).

### Antipsychotics

**FIGURE 5:**
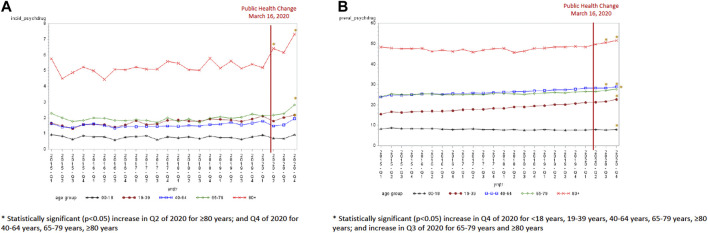
**(A)** Antipsychotic incidence by age group (per 1,000). **(B)** Antipsychotic prevalence by age group (per 1,000).

**FIGURE 6:**
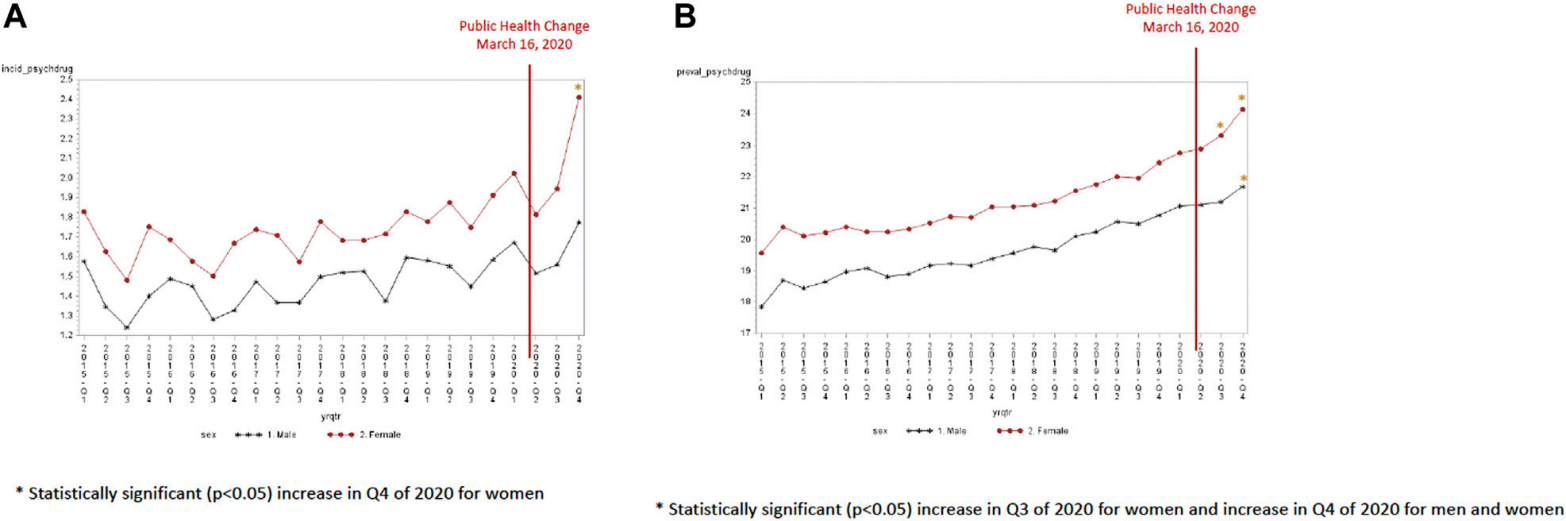
**(A)** Antipsychotic incidence by sex (per 1,000). **(B)** Antipsychotic prevalence by sex (per 1,000).

Overall, an increase in the incidence (1.70/1,000 in Q1:2015 to 1.85/1,000 in Q1:2020) and in the prevalence (18.71/1,000 in Q1:2015 to 21.92/1,000 in Q1:2020) of antipsychotic use was observed from 2015 to 2020 (Supplementary Appendix SIV). The incidence of antipsychotics was highest in the Q4:2020 (2.09/1,000) and the incidence was significantly higher in Q4 of 2020 than the expected trend for those 40 years and older (40 years old: 0.29, SE 0.11, *p* = 0.02; 65–79 years: 0.82, SE 0.18, *p* = 0.0002; ≥80 years old: 1.87, SE 0.38, *p* < 0.0001) (Figure 5A). Antipsychotic incident use was the highest in the 80+ years of age population at 7.33 per 1,000 in the fourth quarter of 2020. The prevalence of antipsychotic use significantly increased for all age groups in quarter 4 of 2020 compared to the expected trend (Figure 5B). A significant increase in antipsychotic prevalence was also observed in quarter 3 of 2020 for those 65–79 years (1.43, SE 0.35, *p* = 0.02) and ≥80 years (2.70, SE 0.99, *p* = 0.013) compared to the expected trend. Females experienced an increase in antipsychotic incidence in quarter 4 of 2020 (2.41 per 1,000) (Figure 6A). In contrast, the incidence of antipsychotic use in the last quarter of 2020 was 1.77 per 1,000 for men. A significant increase in antipsychotic incidence in quarter 4 compared to the expected trend was seen only females (0.52, SE 0.12, *p* = 0.0004). Both males and females experienced an increase in antipsychotic prevalence over time (from 17.84 per 1,000 in the first quarter of 2015 to 21.69 per 1,000 in the last quarter of 2020 for males and 19.57 per 1,000 in the first quarter of 2015 to 24.13 per 1,000 in the last quarter of 2020 for females) (Figure 6B). Females had a significantly higher antipsychotic prevalence in Q3 (0.81, SE 0.28, *p* = 0.01) and Q4 (1.47, SE 0.29, *p* < 0.0001) of 2020. Males had a higher antipsychotic prevalence in Q4 of 2020 (0.52, SE 0.25, *p* = 0.0459).

## Discussion

In this population-based study in the Canadian province of Manitoba, we observed a significant decrease in the incident use of antidepressants and anxiolytics in most age groups and both sexes immediately (i.e., within the second quarter of 2020) following COVID-19 public health measures, compared to the expected trend. We also observed the incidence of antidepressant and antipsychotic use to be the highest at the end of 2020, compared with the same period in the previous 5 years. Women and those aged 40 years and older (especially aged 80 years and older) had the highest incidence in antidepressant and antipsychotic use at the end of 2020.

A surprising finding was an increase in the incidence of antidepressant and antipsychotic use in the final quarter of 2020 across most age groups and both sexes, but particularly in the 80+ year old and female population. A greater increase in the incidence of antipsychotic use among the older adult population is consistent with a previous Canadian study of nursing home residents (Avery et al., 2021). This study from the Canadian province of Ontario found an increase in the mean monthly proportion of nursing home residents receiving a prescription for an antipsychotic, antidepressant, and trazodone in March to September 2020 compared to January to February 2020 (Stall et al., 2021). Prolonged social isolation and reduced availability of nonpharmacological interventions may be contributing factors to these trends. However, it was unexpected to see a similar trend in community-dwelling older adults against national surveys reporting mental health to be less affected by the pandemic than for younger populations (Vahia et al., 2020). Our study also found an increase in incidence in antidepressant and antipsychotic use in the final quarter of 2020 for women and not men. Women may experience particular challenges with prolonged public health restrictions. Home schooling, work demands, child care duties could have an impact on the mental health for women over this period, especially by the last quarter of 2020. Previous studies have cited disproportionate levels of major depressive disorder and anxiety among women, particularly those with children (COVID-19 Mental Disorders Collaborators, 2021; Avery et al., 2021). In Manitoba, the College of Physicians released a Standards of Practice for benzodiazepine prescribing in November 1, 2020, which introduced new requirements and limits on benzodiazepine prescribing (The College of Physicians & Surgeons of Manitoba, 2020). It is possible that these new standards may have resulted in the substitution of anxiolytic/sedative-hypnotics with antipsychotics and/or antidepressants in the last quarter of 2020. It is also not known whether the increase in antidepressant use may be a result of long COVID and care providers may be treating post-COVID depression with antidepressants. One study found individuals who have had COVID-19 experienced greater rates of mental health disorders and antidepressant use than those without COVID-19 (Xie et al., 2022). While the number of reported positive COVID-19 cases in Manitoba were low at the beginning of the pandemic, there were 23,625 Manitobans who had COVID-19 by December 2020, and it is not known what proportion of the population had COVID-19 that was not lab-confirmed (Manitoba Government, 2020a).

The declining trend in both the incidence and prevalence of anxiolytic/sedative-hypnotic use is not surprising considering efforts to minimize the long-term use of these agents (The College of Physicians & Surgeons of Manitoba, 2020). The new Standards of Practice for benzodiazepine prescribing in Manitoba could have affected the prescribing of benzodiazepines in the fourth quarter of 2020 (The College of Physicians & Surgeons of Manitoba, 2020). It is important to also note that we would expect to observe a seasonal trend in which a peak incidence would be observed in quarter 1 (January to March) and a trough incidence would be observed in quarter 2 (April to June) of every year. This is because all eligible Manitoba residents receive full coverage on eligible prescription medications after an income-based deductible is paid, which resets to zero on April 1 of every year. However, at the beginning of the pandemic (quarter 2 or April to June of 2020), we would also expect to see a greater decline in the incidence of psychotropic medication use, and anxiolytic/sedative-hypnotics in particular. This was the period shortly after in-person visits were restricted. It is possible that there may be less comfort among prescribers to prescribe new prescriptions for these agents in a virtual environment or people were less inclined to leave their homes to fill a prescription during the pandemic. While another study also found a significant decline in opioid and benzodiazepine prescriptions following restrictions to elective medical procedures and routine office visits (Downs et al., 2021), there are no studies to support whether a change in comfort in prescribing or having prescriptions refilled in-person is occurring among prescribers and patients, respectively. Most pharmacies in Manitoba offer home delivery or curb-side pick-up of prescriptions. Low incidence of psychotropic medication use in the second quarter could also be explained by a lower priority to initiate care following a major global event. However again there are no studies to support that this is a possibility. Other studies have found an increase in the use of psychotropic medications shortly after a major event (Benjamin and Steven, 2004; DiMaggio et al., 2007).

Drug shortages were a concern in the early months of 2020 (Drug Shortages Canada, 2022). Stockpiling of medication could explain the slight elevation in prevalence of benzodiazepines seen in quarter one. Pharmacists in Manitoba were to provide only a 1-month supply in a 28-day period for all drugs to allow access to medications for patients for medications in short supply. However, this restriction was implemented March 20, 2020 (Manitoba Government, 2020b) and was lifted in May 11, 2020 (Manitoba Government, 2020c). This may explain the fluctuations in prescription fills in quarters 1 and 2 of 2020.

Our findings contrasted the results of a cross-sectional study where the investigators found no clinically meaningful differences in overall prescription rates of psychotropic medications in 2020, compared to 2019, using data from Kaiser Permanente Northern California electronic records (Hirschtritt et al., 2021). After accounting for secular trends or prior year patterns, they found a small, but significant increase in the antidepressant trazodone and mood stabilizers/antipsychotics, and a small decrease in benzodiazepines and hypnotics, with no significant change in antidepressants and stimulants (Hirschtritt et al., 2021). They also found a lower-than-expected trend in new fills for nearly all medications, including antidepressants, benzodiazepines, hypnotics, and mood stabilizers and antipsychotics (Hirschtritt et al., 2021). This was consistent with our findings of a decline in incidence in antidepressants and anxiolytic/sedative-hypnotic use in the second quarter as their data were only limited to the first 13 weeks of the pandemic in California. Their study did not examine the long-term effects of the pandemic on these trends nor did they look back beyond 2019 for secular trends. A major limitation of this study was the use of prescription data from a single insurer, which limits the generalizability to the entire population including those without insurance coverage. This is particularly important during a time when job security may have changed because of the pandemic.

A pilot study by Yu et al. including 365 patients from an independent community pharmacy in North York, Ontario found no difference in the initiation of new prescriptions for antidepressants and antianxiety medications during the first few months of the pandemic compared to the prior year (*p* = 0.251) (Yu et al., 2021). This study did find more frequent dispensing of benzodiazepine tablets (*p* = 0.016) in the first 5 months of 2020 compared to the same period in 2019 (Yu et al., 2021). However, no significant differences were observed in the number of defined daily doses between the two time periods (Yu et al., 2021). This study was limited by its sample size and its data source from one community pharmacy. Uthayakumar et al. similarly found a reduction in antidepressant dispensations in April 2020 but a return to pre-pandemic trends from August to December 2020 (Uthayakumar et al., 2022). This study found no difference in benzodiazepine dispensation before and during COVID-19. This study used data from IQVIA (IMS Health and Quintiles), which captures approximately 78% of prescriptions dispensed in Canada. This study only examined dispensation rates as tablets per 100 population and did not evaluate antipsychotic use.

Strengths of our study included the use of a large administrative database unrestricted by age, income, or insurance coverage. We also examined drug trends to the end of 2020 where previous studies have only examined the first few months of 2020 and we were able to compare to the previous 4 years. Our findings may not be generalizable to populations without universal health care coverage. In addition, DPIN data captures prescriptions received by patients but does not necessarily imply actual consumption of medication. Factors influencing prescription trends are multifactorial (e.g., drug shortages, drug coverage fiscal period, public health restrictions, pandemic) with each factor able to explain the trends observed, therefore it is difficult to pinpoint whether one factor contributed to the observed trends more predominantly than the others. Moreover, the rate of COVID-19 positive patients in Manitoba was low at the beginning of pandemic compared to other jurisdictions during 2020 (273 total COVID-19 positive cases as of April 29, 2020 to 23,625 total number of lab-confirmed cases in Manitoba as of December 24, 2020) (Manitoba Government, 2020a; Manitoba Government, 2020d), and as such it is difficult to generalize findings to locations with higher rates of infection.

This study provided insight on important questions about mental health treatment and consequences related to the pandemic. Findings from this study will help inform decisions around processes of care for mental health.

## Data Availability

The data analyzed in this study is subject to the following licenses/restrictions: Data used in this article was derived from administrative health and social data as a secondary use. The data was provided under specific data sharing agreements only for approved use at MCHP. The original source data is not owned by the researchers or Manitoba Centre for Health Policy (MCHP) and as such cannot be provided to a public repository. The original data source and approval for use has been noted in the acknowledgments of the article. Where necessary, source data specific to this article or project may be reviewed at MCHP with the consent of the original data providers, along with the required privacy and ethical review bodies. Requests to access these datasets should be directed to mchp_access@cpe.umanitoba.ca.
