# Supplementary material for: Psychotropic Medication Use Before and During COVID-19: A Population-Wide Study
Source: Front Pharmacol. 2022 Apr 27;13:886652. doi: 10.3389/fphar.2022.886652 (PMC9092447; doi:10.3389/fphar.2022.886652)

**Appendix I**. Timeline of policy changes in Manitoba

| Date | Description |
| --- | --- |
| March 12, 2020 | First reported COVID-19 Case in Manitoba |
| March 16, 2020 | University closure, childcare services closure, non-essential service operation changes, events consisting of more than 50 people prohibited |
| March 17, 2020 | Advice on dispensing of opioid agonist therapy by CPhM released |
| March 18, 2020 | The College of Pharmacists of Manitoba (CPhM) and the College of Physicians and Surgeons (CPSM) released a guidance document regarding safe access to Manitoba Prescribing Practices Program (M3P) prescriptions for patients during the pandemic in which all M3P prescriptions may temporarily be faxed from prescribers directly to the pharmacy. |
| March 19, 2020 | Pharmacists in Manitoba may only provide a one-month (to a maximum of 35 days) supply in a 28-day period for all drugs (MHSAL announcement). |
| March 19, 2020 | Health Canada issued a short-term exemption under the Controlled Drugs and Substances Act (CDSA) permitting pharmacists to extend or transfer CDSA prescriptions, and accept verbal orders if permitted within the applicable provincial or territorial scopes of practice during the COVID-19 pandemic. (Not in place in Manitoba) |
| March 20, 2020 | State of emergency declared for 30 days |
| May 11, 2020 | Prescription restriction of one month lifted |

**Appendix II**. ICD Codes used to define psychiatric disorders^19-24^

| Disorder | ICD-9-CM | ICD-10CA |
| --- | --- | --- |
| Mood and/or anxiety | 296, 300, 300.2, 300.3, 300.7, 300.4, 309, 311 | F31-34.1, F38, F38.1, F40, F41, F41.1, F41.2, F41.3, F41.8, F41.9, F42, F43.1, F43.2, F43.8, F45.2, F53, F68, F93, F99 |
| Psychosis | 295-299 | F2, F3, F84, F410 |
| Schizophrenia | 295 | F20, F21, F23.2, F25 |
| Personality disorder | 301 | F21, F34.0, F60, F62, F68.1, F68.8 or F69 |
| Substance use disorder | 291, 292, 303, 304, 305 | F10-F19, F55 |

**Appendix IV**. Figures on quarterly prevalence and incidence of antidepressants, sedative-hypnotic/anxiolytic, and antipsychotics for the general population

**SuppFigure 1.** Quarterly incidence of antidepressants (per 1000) from 2015 to 2020


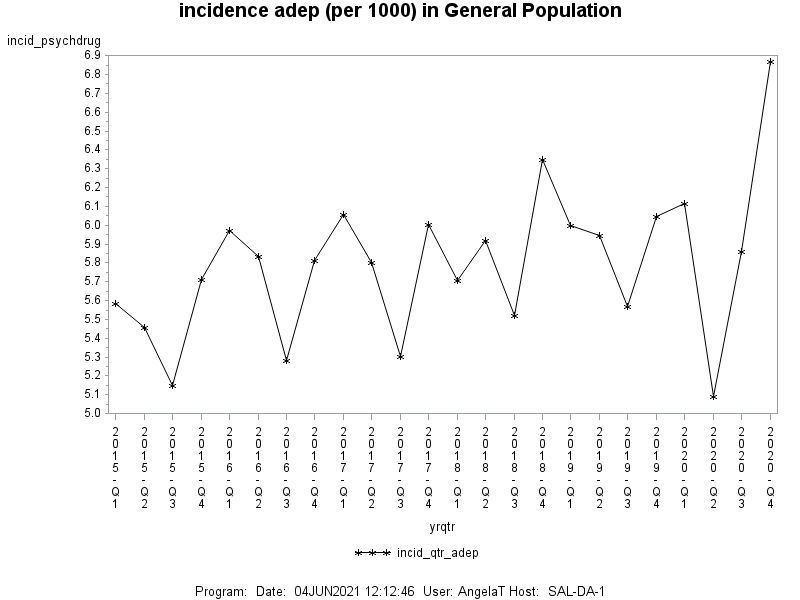


**SuppFigure 2.** Quarterly prevalence of antidepressants (per 1000) from 2015 to 2020


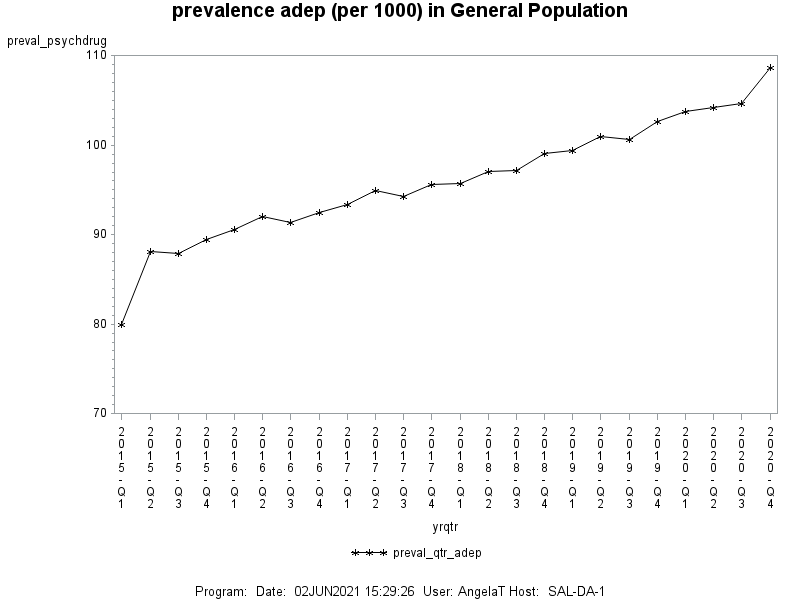


**SuppFigure 3.** Quarterly incidence of anxiolytic/sedative-hypnotic (per 1000) from 2015 to 2020


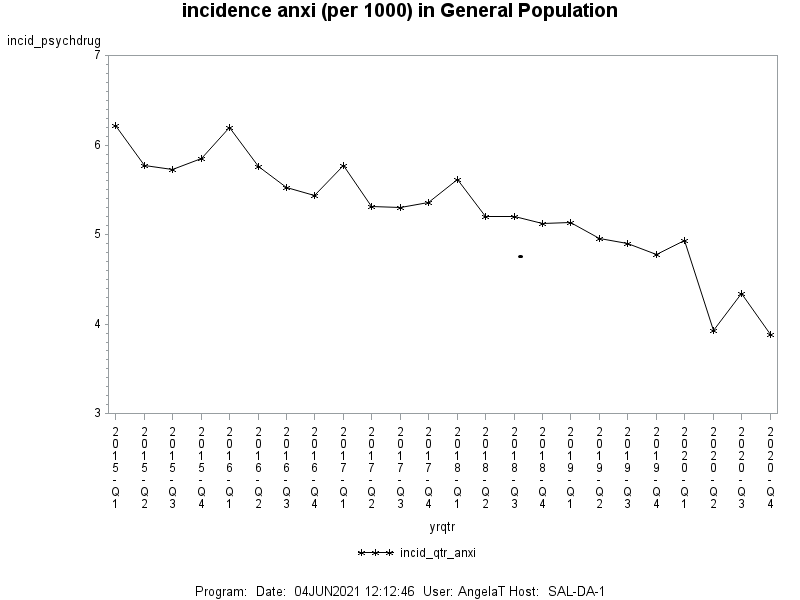


**SuppFigure 4.** Quarterly prevalence of anxiolytic/sedative-hypnotic (per 1000) from 2015 to 2020


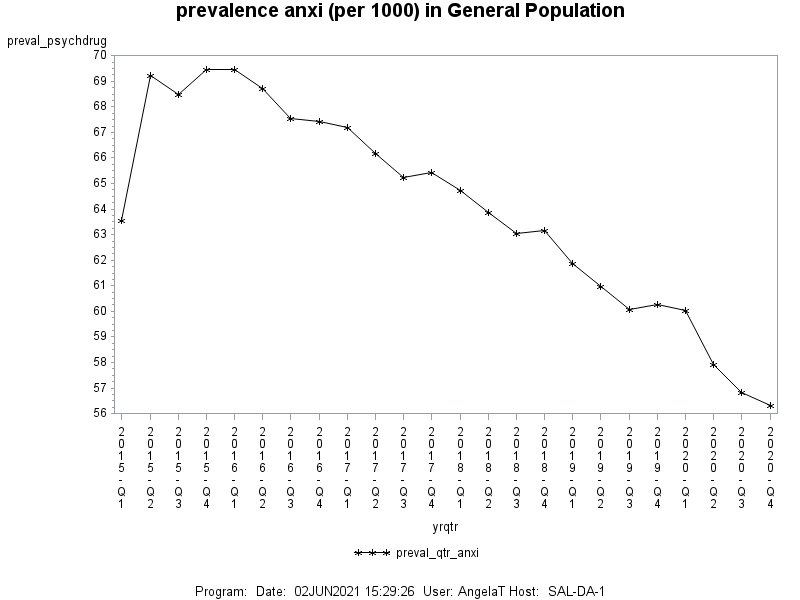


**SuppFigure 5.** Quarterly incidence of antipsychotics (per 1000) from 2015 to 2020


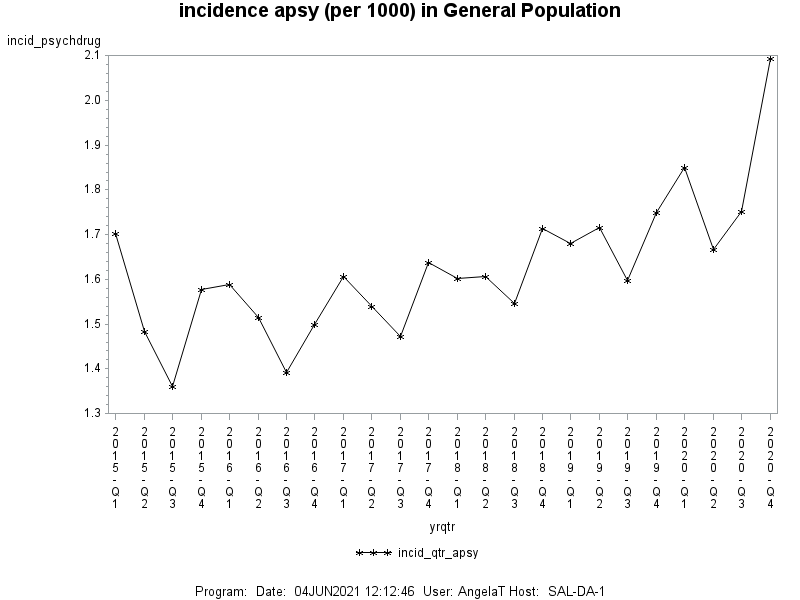


**SuppFigure 6.** Quarterly prevalence of antipsychotics (per 1000) from 2015 to 2020


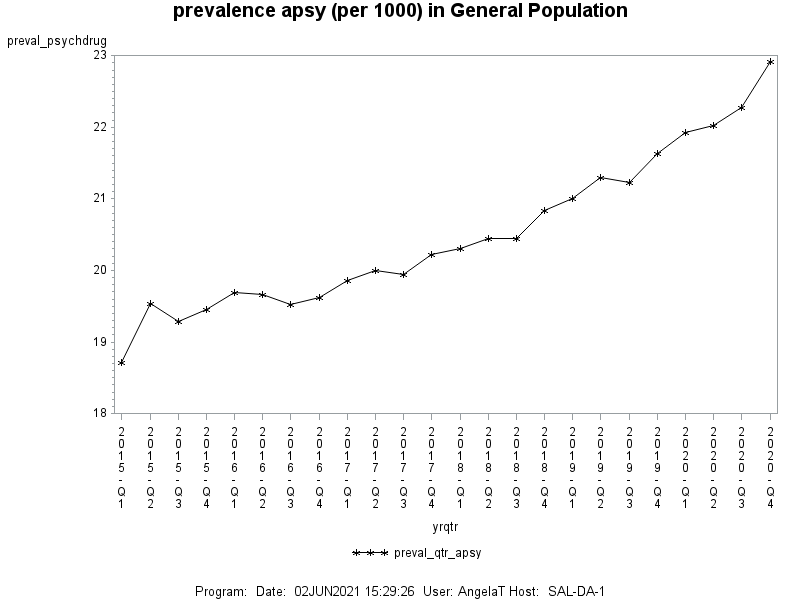

Supplement: Supplementary file 1 [file DataSheet1.docx]
